# Safety and efficacy of miltefosine in cutaneous leishmaniasis: An open label, non-comparative study from Balochistan

**DOI:** 10.12669/pjms.35.2.54

**Published:** 2019

**Authors:** Moizza Tahir, Uzma Bashir, Javeria Hafeez, Rabia Ghafoor

**Affiliations:** 1 *Dr. Moizza Tahir, MCPS (Med), FCPS (Derm), MHPE, Associate Professor, Department of Dermatology, Combined Military Hospital/CMH Institute of Medical Sciences, Multan, Pakistan*; 2 *Dr. Uzma Bashir, MCPS(Med), FCPS(Derm), Assistant Professor of Dermatology, Combined Military Hospital/Quetta Institute of Medical Sciences, Quetta, Pakistan*; 3 *Dr. Javeria Hafeez, MCPS(Med), FCPS(Derm), Assistant Professor of Dermatology Combined Military Hospital, Bhawalpur, Pakistan*; 4 *Dr. Rabia Ghafoor, FCPS(Derm), SCE(UK), Assistant Professor of Dermatology, Jinnah Post Graduate of Medical Sciences, Karachi, Pakistan*

**Keywords:** Cutaneous leishmaniasis, Meglumine antimoniate, Miltefosine

## Abstract

**Background & Objective:**

Cutaneous Leishmaniasis (CL) is endemic in Baluchistan and treated traditionally with Meglumine antimoniate. Miltefosine appears appealing therapy in cutaneous Leishmaniasis. Our objective was to evaluate safety and efficacy of Miltifossine in treatment of cutaneous Leishmaniasis.

**Methods:**

This experimental study was conducted from 10 September 2017 to 10 May 2018 at Combined Military Hospital Quetta. Total of 42 patients were recruited by purposive sampling technique. Lesional skin smears were stained with giemsa for Leishmania amastigotes under magnification (100 x).Complete blood count, serum urea, creatinine, bilirubin, aspartate aminotransferases (AST), alanine aminotransferase (ALT) were done at the beginning of treatment and then weekly, thereafter. Cap Miltefosine 50 mg (2.5mg/kg) were given as directly observed therapy .Daily observation during treatment phase was done for clinical side effects of therapy. Clinical response was documented at two weeks then at eight weeks. Photographs were taken before and after the therapy. Data was analyzed by SPSS 16.

**Results:**

Complete clinical response was observed in 39 (92.9%) patients and partial clinical response in 1(2.4%) patient. Two patients were lost to follow up at eight weeks. No significant derangements in laboratory profile were noted before and after treatment. Mean duration of treatment was 23.47+SD 4.44 days. Sixteen patients (38.1%) took Miltefosine for 28 days, 12 (28.6%) for 21 days and 9 (25%) for 25 days.

**Conclusion:**

Miltefosine is safe and cost effective treatment for cutaneous Leishmaniasis. It is effective in CL cases not susceptible to antimony compounds.

## INTRODUCTION

Leishmaniasis is a vector-borne disease, caused by protozoan parasite of the genus Leishmania. Over 12 million individuals get infection across the globe and WHO reports over two million new cases each year.^1^ CL and MCL (mucocutaneous Leishmaniasis) are predominantly diagnosed in Afghanistan, Algeria, Colombia, Brazil, Iran and additional African and Latin countries.^2^ CL is endemic in Balochistan, Khyber Pakhtunkhwa, Punjab and Sindh.^1^,^3^ The infection is seen in young and non-immune adults. The epidemiological pattern of the disease is similar to the neighboring areas of Iran, Afghanistan and Rajasthan in India.^4^

Sand fly Phlebotomus spp in Europe, North Africa, Middle East, and Asia or of the Lutzomyia spp in the Southern USA to Northern Argentina are vectors of disease.^1^ Clinical manifestations are cutaneous, mucocutaneous and visceral Leishmaniasis. It is influenced by host factors such as genetic and immunological status and parasite virulence which varies with type of species. Localized cutaneous Leishmaniasis (LCL) is caused by L. major, L. tropica, L. infantum, and L. aethiopica in the old world. New world Leishmaniasis is caused by L. braziliensis, and in Africa, L. aethiopica. Self-healing sores of Leishmania major heal within 3–9 month, where as in case of Leishmania tropica infection heal within 6–15 months.^1^

Pentavalent antimony compounds are considered as gold standard in the treatment of Leishmaniasis. However drug resistance has been increasingly documented with a cure rate not beyond 80%.^5^ Resistance to antimonials lead to treatment failure in particular to anthroponotic cutaneous leishmaniasis.^6^ Miltefosine appears promising in the treatment of cutaneous leishmaniasis.^7^ It has been approved by FDA for infections by Leishmania (V.) braziliensis, L. (V.) panamensis, and L. (V.) guyanensis.^7^ Miltefosine therapy for old World species is its off-label use. Its efficacy has been found similar to Meglumine antimoniate.^2^ However limited data has been available.^8^

Effectiveness of miltefosine also varies in different geographic regions^9^,^10^. However, there are a number of limitations with these drugs including cost, efficacy, adverse effects and duration of treatment. Considering the epidemiological impact of Leishmaniasis, development of safe, effective and affordable treatment option remains a concern.^2^,^11^

Our study targeted population at combined military hospital Quetta which is a tertiary care center for military in particular and for civilian population from remote areas of Baluchistan. Cutaneous Leishmaniasis resistant to meglumine antimoniate is not infrequent. The aim of our study was to evaluate efficacy and safety of miltefosine in the treatment for cutaneous Leishmaniasis. The study would inform practice.

### Abbreviations Used

**CL** Cutaneous Leishmaniasis

**MCL** Mucocutaneous Leishmaniasis

**FDA** Food and Drug Administration

**AST** Aspartate aminotransferases

**ALT** Alanine aminotransferase

**VL** Visceral Leishmaniasis

**LD** Leishmania Donovinai

## METHODS

This was experimental study conducted from 10 September 2017 to 10 May 2018. Total of 42 patients was recruited by purposive sampling technique. An inclusion criterion were patient with a lesion consistent with clinical diagnosis of cutaneous Leishmaniasis with positive LD smear on giemsa stain but did not respond to parenteral Meglumine antimoniate in past three months, or were intolerant to parenteral Meglumine antimoniate as demonstrated by toxic side effects such as incapacitating myalgias, raised ALT to more than four times the baseline during therapy, drug fever. Patients with hypersensitivity to meglumine antimoniate as demonstrated by intra dermal test dose reaction within 48 to 72 hours. Patient positive for HCV, Hep B or acute or chronic illness, pregnant females, nursing mothers and children under 12 years were excluded.

Patients were enrolled after physical and cutaneous examination. Lesional skin smears were stained with giemsa. Infection was confirmed by identification of Leishmania amastigotes under magnification (100 x). Complete blood count, serum urea, creatinine, bilirubin, aspartate aminotransferases (AST), alanine aminotransferase (ALT) were done at the beginning of treatment and then weekly, thereafter. Daily observation during treatment phase and follow up visit at two weeks, three weeks and then finally at eight weeks was done.

Capsule Miltefosine (Cap Fosine by Nimrall pharma) was administered in a dose of 2.5mg/kg daily to patients. Capsules are available in strength of 50 mg and adult of 70 kg received 50 mg three times a day. Patients were observed for complaints of nausea, vomiting, diarrhea, fever or any other discomfort.

Patient’s lesion was observed for clinical response at two weeks, three weeks and then at eight weeks by another dermatologist working in dermatology department. Complete clinical response was defined as 100% reepithelisation; partial clinical response was defined as regression of lesion with reepithelisation 75%-99%, clinical failure was defined as no regression in lesion. Clinical relapse is defined as development of new lesion at original site or enlargement of previous lesion showing partial response. Photographs were taken to document clinical response. Ethical approval was taken from institutional ethical review committee. SPSS version 16 was used to analyze data. The descriptive analysis included simple frequencies and the median, mean and standard deviation, whenever appropriate.

**Fig.1:**
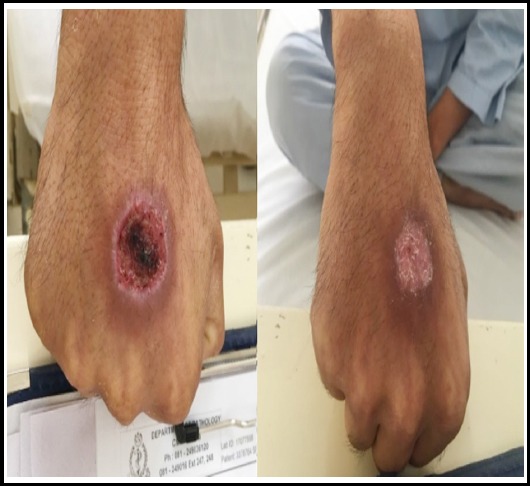
Ulcer hand at 2 Weeks / 8 Weeks.

## RESULTS

A total of 42 patients (40 male and 2 females) were enrolled in eight months of the study period after their informed consent. The age of patient ranged from 20 to 51 years (Median 30. 35 years).Two females were house wives, 35 males were serving soldiers, one was night security guard, and four were self-employed. The average number of lesions per patient was 1.7. Of all the lesions 47.6% (20) were located on ankles, 38.1% (16) on legs, 9.5% (4) on arms and 4.8% (2) on face. Smear for amastigotes were positive in 39 (92.9%) and negative in 3(7%) patients. Baseline serum biochemistry and blood counts were normal in all patients. Nausea and vomiting was observed in seven patients, diarrhea in two, serum aminotransferases and transaminase were raised in one patient on seventh day of treatment. Skin rash developed in one patient on eighth day, and settled on 13th day. Discontinuation of therapy was done for three to four days and then resumed.

**Fig.2:**
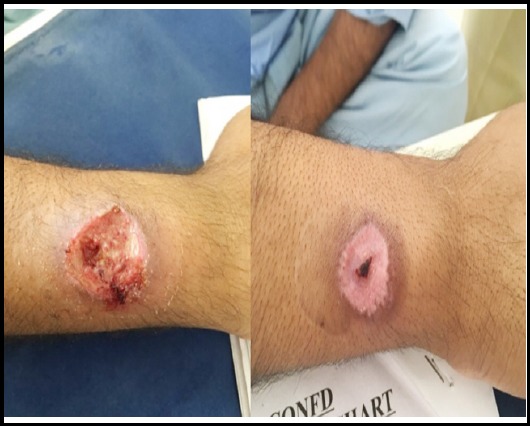
Ulcer hand at 2 Weeks / 8 Weeks.

**Fig.3:**
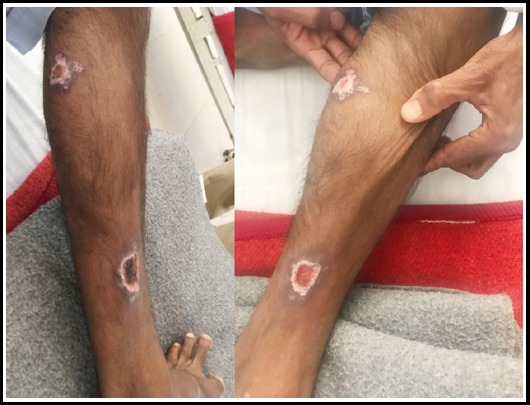
Ulcer Leg at 2 Weeks / 4 Weeks

**Fig.4:**
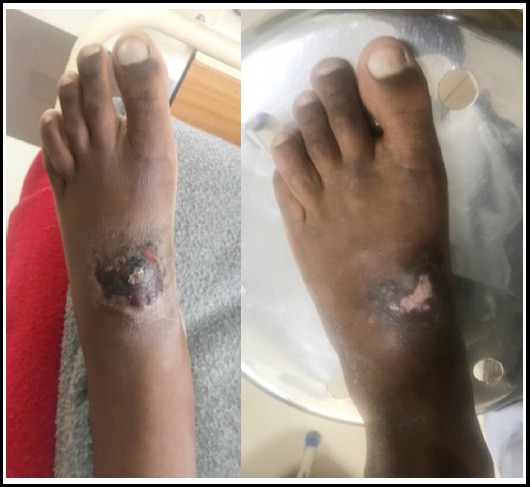
Ulcer Leg at 2 Weeks / 8 Weeks.

**Fig.5:**
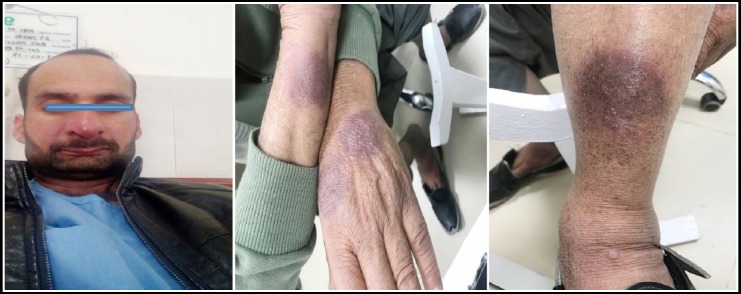
Complete clinical response at 8 weeks.

Complete clinical response was observed in 39 (92.9%) patients, whereas partial clinical response in 1 (2.4%) patient at 08 weeks. Two patients were lost to follow up. None of our patient had clinical failure or relapse at 08 weeks. Mean duration of treatment was 23.47±SD 4.44 days. Sixteen patient (38.1%) took Miltefosine for 28 days, 12 (28.6%) for 21 days and 9 (4%) for 25 days.

## DISCUSSION

Cutaneous Leishmaniasis is a major public health concern. Clinical features range from self-healing sores to chronic disfiguring disease. No vaccine has been developed for Leishmaniasis, and drug therapy is often ineffective.^12^ Advancements in technology have allowed testing compounds against Leishmania parasites, although no new drugs have been registered so far. Antimony compounds remain traditional treatment of choice for Leishmaniasis. Parenteral and intralesional Meglumine antimonite are first line of treatment.^13^ Concerns about antimoniate safety and efficacy are questionable.^14^ In 2014, oral agent Miltefosine had been approved by FDA for cutaneous, mucosal, and visceral Leishmaniasis.^15^

Miltefosine is an alkylphosphocholine drug. It is active against various parasites, pathogenic bacteria, fungi and cancer cells. It is the only oral drug that can be used to treat VL and CL.^16^ Capsule Miltefosine in monotherapy regimen of 2.5mg/kg body weight is well tolerated for 28 days. Our patients developed gastrointestinal side effects but they were well tolerated. This finding is consistent with other studies.^16^

Complete clinical response was documented in 92.9% of our patients with average duration of treatment of 23.47±SD4.4 days. When comparing monotherapy of Miltefosine with Meglumine antimoniate (15ml I/M or IV)) in Pakistani context, Miltefosine therapy is more cost-effective. WHO negotiated price of cap Miltefosine for adult is 45.28$ for 56 capsule^17^ (1 cap is of 50mg and recommended dose is 50 mg TDS).Complete Miltefosine course of 28 days will be of 67.2$. WHO negotiated price of Meglumine antimoniate (Glucantime, Aventis)is 1.2 $/5ml vial of 81mg/ml. Recommended dose is 14ml so 28 day cost will be 106.4$. Meglumine antimoniate will additionally include indirect cost (i.e. loss of productivity by hospital admission, monitoring of toxic effects), prolonged hospital stay as the injections need to be withheld if labs are deranging. Additionally as monotherapeutic option, Miltefosine appeared to be the cost-effective option in areas where there is known resistance to antimony compounds.

None of our treated case of cutaneous Leishmaniasis relapsed at two month follow up visit. Decrease in the susceptibility of Leishmania parasites to miltefosine in vivo has not been documented. This decrease in susceptibility is a precursor of the emergence of drug resistance. However relapses after successful primary miltefosine treatment in immunocompetent patients have been reported for both CL and VL.^16^,^18^

In our study males are affected more than females. This is due to their greater indulgence in field activities. Majority of our patients were soldiers. These findings are consistent with other studies as occupations that require staying outdoors for a part of night, e.g., soldiers and security guards place them at greater risk.^19^ Our findings showed that males were more affected than females (P=0.04 These patients had lesions on upper and lower limbs, a finding consistent with other studies.^19^

### Limitations of study

Leishmania spp identification was not done due to non-availability of facility. Follow up of cases was up to 08 weeks as most of our patients were soldiers deployed at remote areas. Their access to hospital was a technical limitation.

## CONCLUSION

Miltefosine is an oral, safe and effective treatment for cutaneous Leishmaniasis. It is effective in CL cases not susceptible to antimony compounds.
